# Use of shoulder pacemaker for treatment of functional shoulder instability: Proof of concept

**DOI:** 10.1007/s11678-017-0399-z

**Published:** 2017-04-04

**Authors:** Philipp Moroder, Marvin Minkus, Elisabeth Böhm, Victor Danzinger, Christian Gerhardt, Markus Scheibel

**Affiliations:** Department of Shoulder and Elbow Surgery, Center for Musculoskeletal Surgery, Charité – Universitätsmedizin Berlin, Augustenburger Platz 1, 13353 Berlin, Germany

**Keywords:** Shoulder Pacemaker, Functional shoulder instability, Electrical stimulation, Posterior shoulder instability, Muscle patterning, Schulterschrittmacher, Funktionelle Schulterinstabilität, Elektrostimulation, Hintere Schulterinstabilität, Polar Typ 3 Instabilität

## Abstract

**Background:**

Functional shoulder instability (polar type III) is caused by underactivity of rotator cuff and periscapular muscles, which leads to subluxation or dislocation during shoulder movement. While surgical treatment has shown no benefits, aggravates pain, and frequently diminishes function even further, conservative treatment is often ineffective as well.

**Objectives:**

The aim was to investigate the effectiveness of a “shoulder pacemaker” device that stimulates underactive muscles in patients with functional instability during shoulder movement in order to re-establish glenohumeral stability.

**Patients and methods:**

Three patients with unsuccessfully treated functional shoulder instability causing pain, emotional stress, as well as limitations during daily activities and sports participation were enrolled in this pilot project. The device was used to stimulate the external rotators of the shoulder and retractors of the scapula. Pain level, subjective shoulder instability, range of motion, visible aberrant muscle activation, and signs of dislocation were compared when the device was switched on and off.

**Results:**

No changes were observed when the device was attached but switched off. When the device was switched on, all patients were able to move their arms freely without pain, discomfort, or subjective or objective signs of instability. All patients rated this as an excellent experience and volunteered to train further with the device. No complications were observed.

**Conclusion:**

The electric stimulation of hypoactive rotator cuff and periscapular muscles by means of the shoulder pacemaker successfully re-establishes stability in patients with functional shoulder instability during the time of application.

****Video online**:**

The online version of this article (doi: 10.1007/s11678-017-0399-z) contains the video: “The Shoulder-Pacemaker: treatment of functional shoulder instability with pathological muscle activation pattern”. Video by courtesy of P. Moroder, M. Minkus, E. Böhm, V. Danzinger, C. Gerhardt and M. Scheibel, Charité Universitätsmedizin Berlin 2017, all rights reserved

## Zusammenfassung

**Hintergrund:**

Funktionelle Schulterinstabilität (Polar-Typ III) wird durch eine Unterfunktion der Rotatorenmanschette und periskapulären Muskulatur verursacht. Durch chirurgische Eingriffe lässt sich oft keine Besserung erzielen, sondern stattdessen verschlechtern sich Schmerzen und Funktion zusätzlich. Doch auch die konservative Therapie zeigt häufig keinen Erfolg.

**Ziel der Arbeit:**

Ziel dieses Pilotprojekts war es, die Wirksamkeit des „Schulterschrittmachers“ zu untersuchen. Dieser stimuliert hypoaktive Muskelgruppen, um die glenohumerale Stabilität bei funktioneller Instabilität wiederherzustellen.

**Methoden:**

Es wurden 3 Patientinnen mit therapierefraktärer funktioneller Schulterinstabilität, die Schmerzen, Unbehagen, Stress sowie Einschränkungen im Alltag und Sport verursachte, in die Studie eingeschlossen. Durch den Schulterschrittmacher wurde eine Stimulation der Außenrotatoren und der skapularetrahierenden Muskulatur erreicht. Schmerz und Bewegungsausmaß sowie subjektive und objektive Schulterstabilität wurden bei ein- und ausgeschaltetem Gerät analysiert.

**Ergebnisse:**

Keine Änderung der Beschwerden konnte bei inaktivem Gerät festgestellt werden. Eine freie Beweglichkeit ohne Schmerzen, Unbehagen, subjektive oder objektive Instabilität war bei aktiviertem Gerät möglich. Alle Patientinnen bewerteten dies als positive Erfahrung und meldeten sich freiwillig zum weiteren Training mit dem Gerät. Komplikationen wurden keine beobachtet.

**Schlussfolgerung:**

Der Schulterschrittmacher ist durch Stimulation hypoaktiver Muskelgruppen in der Lage, während der Dauer der Anwendung die Stabilität bei Patienten mit funktioneller Schulterinstabilität erfolgreich wiederherzustellen.

## Introduction

Shoulder instability can best be described as an excessive translation of the humeral head in relation to the glenoid, which is responsible for symptoms in the conscious patient [24]. This implies that patients can report various functional symptoms, ranging from a feeling of instability or a loose shoulder to pain during certain movements or even loss of range of motion. The complexity of glenohumeral instability is also represented in its various classification systems. The Stanmore classification of shoulder instability is an easy yet comprehensive classification that distinguishes three different groups according to the respective cause of instability [7]:

- Polar type I patients have suffered a trauma with resultant structural damage to the glenohumeral joint, which leads to shoulder instability.
- Polar type II instability patients show a constitutional deficit such as capsular insufficiency or reduced concavity of the glenoid surface [18, 25], which predisposes these patients to shoulder instability without the necessity of a relevant trauma.
- Polar type III describes a type of instability that is not generated by structural defects but rather caused by an aberrant activation pattern of rotator cuff and periscapular muscles [7].

Several studies confirmed that inappropriate muscle activation patterns can contribute or lead to glenohumeral instability [1, 2, 4, 6, 11, 12, 13, 15, 16, 19, 21, 22]. The humeral head translates excessively, provoking a subluxation or dislocation every time the shoulder passes a particular phase of movement [23]. Common symptoms reported are pain during movement of the arm, a loss in range of motion due to weakness or blockage that inhibits any further movement, as well as a strong feeling of instability that extensively limits shoulder function. The majority of patients with a muscle-patterning instability show an excessive posterior translation of the humeral head [14, 23] representing a functional dynamic posterior shoulde instability (B1) according to the ABC classification [17].

Studies have shown that patients with an abnormal muscle pattern and resultant posterior shoulder instability demonstrate underactivity of the external rotators and scapula retracting muscles including the infraspinatus, lower trapezius, serratus anterior, and posterior deltoid [7, 23]. Physiologically, the humeral head is centered by the rotator cuff and periscapular muscles during movement, thus preventing subluxation or dislocation. However, when the external rotators are hypoactive during an elevation of the arm while the internal rotators including the latissimus dorsi and pectoralis major and the anterior deltoid show a normal or even increased activity, a force disbalance is provoked that makes the humeral head translate posteriorly. This translation is associated with pain, a restricted movement, and/or posterior dislocation [7, 23]. Additionally, these patients often present with altered scapular motion pattern and impairment of the scapulothoracic rhythm [9, 10, 20].

Most of these patients are adolescent and suffer from this condition for an extensive period of time. Not seldom the patients visit several doctors before the correct diagnosis is made and often the ensuing conservative treatment is of no or limited success. In addition to limitations during sports or even activities of daily living, these patients frequently experience emotional stress and are stigmatized by their peers because of their condition. It is generally accepted among shoulder surgeons that surgical treatment is not indicated [7, 8, 23]. However, in our clinical practice we sometimes encounter patients with functional instability who have undergone several surgical stabilization attempts due to misdiagnosis with often catastrophic outcome.

While some success in conservatively treating these patients has been reported from highly specialized physiotherapy units, in our experience, regular physical therapy and muscle training therapy commonly available often do not lead to the desired outcome. Therefore, our goal was to invent a so-called shoulder pacemaker, which stimulates hypoactive muscles during shoulder movement in patients with functional instability in order to re-establish the muscular balance and prevent abnormal translation and consecutive dislocation of the humeral head.

## Methods

### Patients

From September to October 2016, three female patients aged 15, 17, and 21 years who presented with functional shoulder instability were enrolled in this pilot project after obtaining informed consent. For the two underage patients, informed consent was obtained from the parents as well. All patients showed involuntary functional dynamic instability of the dominant shoulder. The initial instability episode occurred as a result of a minor trauma in two patients, while the third patient reported atraumatic recurrent shoulder subluxations since her early childhood. In all patients shoulder function was severely impaired making them reduce or completely pause their physical activities. One patient had a previous arthroscopic surgical stabilization attempt and all three completed physiotherapy for a minimal period of 6 months without a satisfactory improvement of their symptoms. No relevant pathological changes of the soft tissue and bony structures were observed in any of the shoulders of the three patients on the available current magnetic resonance imaging (MRI) scans except for a flat glenoid articular surface in patient number 2.

### Technical background

Before electrical muscle stimulation was applied on patients, the author (P.M.) tested the effect on different muscles as well as the required electric current in a self-experiment. Inadvertent sudden muscle contractions or pain can be avoided by raising the current carefully.

**Fig. 1:**
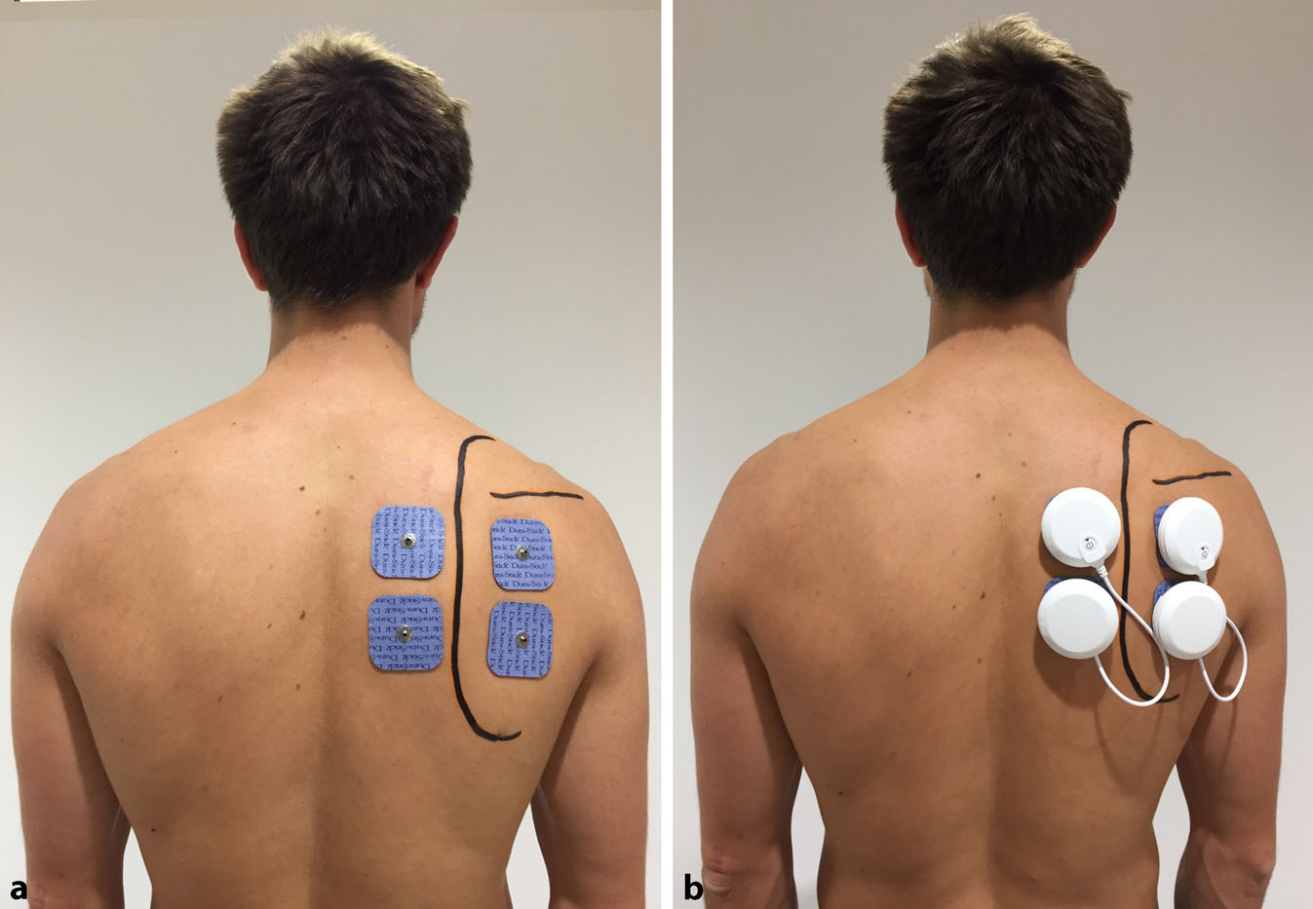
Placement of the shoulder pacemaker electrodes to stimulate external rotators and scapula retractors

Two electrodes were placed medial to the medial border of the scapula, activating the muscles that stabilize the scapula to the thorax (musculi rhomboidei and m. trapezius). Another two electrodes were positioned lateral to the medial border of the scapula inferior to the spinae scapulae, aiming to activate the infraspinatus muscle and posterior deltoid (Fig. 1). After correctly placing the four electrodes, an electric current was applied and increased until the patient felt a tonic muscle contraction. The intensity of the stimulatory energy was carefully increased to a level as high as the individual patient could tolerate without feeling any discomfort. The device utilized for this project was the Chattanooga Wireless Professional muscle stimulator (DJO Global, Vista, Calif.). It comprises four separate stimulation modules that are regulated with a wireless remote. Electric muscle stimulation is a technique during which a transcutaneous electric current is applied to initiate an action potential in electrically excitable cells including nerve and muscle cells. A rectangular compensated current is thereby used to achieve the required threshold potential for the induction of an action potential and consequently a nerve impulse or muscle contraction. The electric parameters of this kind of current are minimal and the required energy for stimulation is low, making the application more pleasant for the patient. The electric stimulation was conducted with a frequency of 35 Hz and a constant current for a pulse duration of 4 s in order to achieve a tonic contraction of the muscle fibers. The patient was then asked to elevate the arm slowly. During elevation, the previously determined electric current triggering a muscle contraction was applied. After one cycle, the current was paused for a few seconds before the next electric stimulation was performed. Pain level, subjective feeling of shoulder instability, range of motion, as well as macroscopically visible aberrant muscle activation pattern and changes in the shoulder contour as a sign of dislocation were assessed and compared when the stimulator was switched on and off.

## Results

### Patient 1

Patient 1 was a 15-year-old student, who experienced an initial instability episode at the age of 12 years as a result of a minor trauma while dancing. She reported a feeling of severe involuntary instability with associated pain (VAS = 7) that required intermittent use of pain medication, and a highly limited shoulder function during sports and activities of daily living. The subjective shoulder value (SSV), the patient’s estimation of the value of the affected shoulder as a percentage of that of an entirely normal shoulder [3], amounted to 30%. The patient experienced instability during each elevation motion of the arm as well as less frequent temporary motion blockades. Clinical examination at our hospital revealed an aberrant movement pattern with pronounced scapular dyskinesia and what appeared to be a temporary scapulothoracic blockade leading to the patient- and observer-perceived impression of posterior glenohumeral dislocation. The range of motion was significantly limited to a flexion of 75°. Despite the absence of structural damage, she had previously been treated surgically with an arthroscopic anterior and posterior capsular plication in another hospital as a salvage attempt but continued to suffer from severe instability while elevating her arm. No subjective improvement was achieved during 1 year of physiotherapy.

**Fig. 2:**
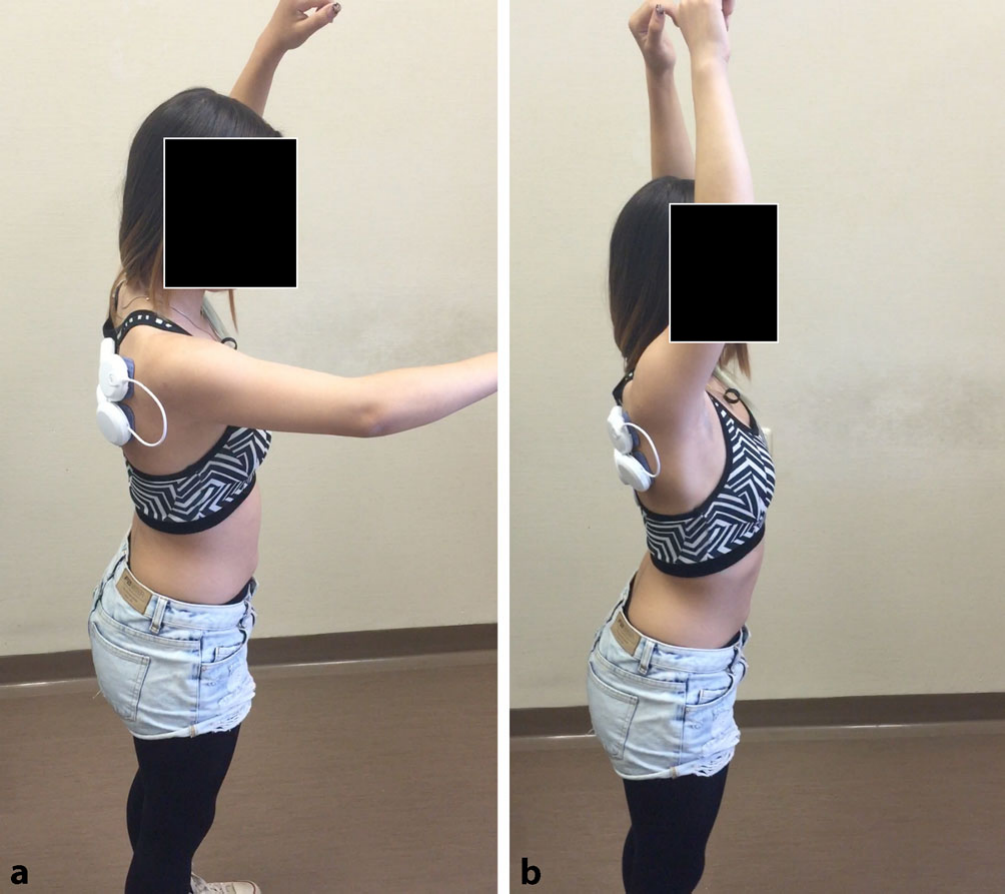
Shoulder flexion capacity of patient 1 when the shoulder pacemaker was switched off (**a**) and on (**b**)

The targeted electric stimulation successfully corrected the muscular imbalance present in this patient. Using the shoulder pacemaker, the patient was able to fully elevate her arm 170° without instability or blockage (Fig. 2). The stimulation of the previously underactive muscles of the shoulder girdle and rotator cuff seemed to effectively stabilize the shoulder. The patient was relieved of the associated pain and was able to move her arm without discomfort in all degrees of freedom (Video 1).

### Patient 2

Patient 2 was a 21-ear-old university student, who suffered from recurrent atraumatic subluxations since her early childhood. The subluxations were caused by elevation of the arm and were not associated with pain (VAS = 0) or a restriction of range of motion. However, the patient complained of severe instability with impaired function during activities of daily living and sports, with a reported SSV of 30%, especially because of a subjective deficit in strength as well as a high resultant emotional stress. Clinically, a functional dynamic posterior shoulder instability with marked scapular dyskinesis could be observed. Previous treatment consisted of several months of physiotherapy over a number of years without a subjective benefit.

Using the shoulder pacemaker, the aberrant muscle activation pattern during elevation of the arm was successfully corrected. The patient was able to move her arm freely in all degrees of freedom, including a flexion of 180° without scapular dyskinesia, subluxations, or an accompanying feeling of shoulder instability. Although the symptoms were already present for a long period, the patient reported increased confidence with her shoulder during the movement of her arm under simultaneous electric stimulation resulting from the conceived improved muscular stabilization.

### Patient 3

Patient 3 was a 17-year-old student, who experienced recurrent subluxations since an initial questionably traumatic subluxation during a handball game at the age of 13 years. Clinical examination revealed a functional dynamic instability with postero-inferior subluxations that occurred at a flexion angle of 90° and were associated with pain (VAS = 4) as well as a slight scapular dyskinesis. The range of motion was not significantly restricted and the patient was able to actively elevate the arm beyond the point of subluxation when bearing the relatively high discomfort. The limitation during over-head movements hindered the patient in her activities of daily living and sports with an SSV of 40%. The patient regularly completed physiotherapeutic treatment over a period of 1 year, but did not experience any improvement in her symptoms.

Electric stimulation of the underactive shoulder muscles successfully confined the pathologically increased translation of the humeral head with a respective clinical improvement. The patient was consequently able to elevate her arm to a level of 170° without subluxation. When the electric current was applied, she reported the previous feeling of instability and discomfort during an elevation of the arm to be completely abolished.

The effect of the shoulder pacemaker in all three cases is displayed in Video 1.

## Discussion

During this pilot study, electric stimulation of underactive external rotators and periscapular muscles using a muscle stimulator was tested in three patients for the treatment of functional shoulder instability. All three patients suffered from extensive functional impairment as well as high emotional stress resulting from the recurrent involuntary instability episodes experienced during arm movement and mostly unsuccessful previous conservative and surgical treatments. The targeted electric muscle stimulation enabled all patients to freely move the arm in all degrees of freedom without signs of instability and accompanying discomfort. All three patients were greatly relieved and excited to see their shoulder instability disappear at least for a short time. No complications were observed or reported during or after the treatment.

There is typically no structural damage present in patients with muscle-patterning shoulder instability and surgical treatment is therefore contraindicated. In 1984, Hawkins et al. published a retrospective study of 50 shoulders in 35 patients with mostly voluntary (functional) recurrent posterior instability [5]. In this cohort, 26 shoulders were treated surgically with a posterior glenoid osteotomy (*n* = 17), posterior plication (*n* = 6), or biceps transfer (*n* = 3). The remaining 24 shoulders were treated nonoperatively. Patients achieved generally poor results, particularly after operative treatment. After surgical reconstruction, the recurrence rate was 50% and in five cases complications led to revision surgery. In the conservatively treated group the symptoms improved but were still present. The authors concluded that patients suffering from recurrent posterior shoulder instability with voluntary uni- or bilateral dislocations should be treated conservatively by appropriate rotational strengthening and surgery should be avoided [5].

In 1973, Rowe et al. presented a case series of 26 patients with voluntary (functional) uni- or bilateral shoulder dislocations [22]. Electromyographic examinations demonstrated that dislocation of the humeral head was provoked by a selective partial suppression of the muscle force-couples responsible for shoulder motion [22]. The authors found that most patients responded well to resistive exercises to strengthen the abductors as well as internal and external rotator muscles. Additionally, psychological disorders were commonly observed and mentioned to interfere with the treatment success. Surgical treatment for these patients may worsen the symptoms owing to possible complications and lead to poor results [22].

Jaggi et al. performed dynamic electromyographic analysis in patients with recurrent shoulder instability [8]. The latissimus dorsi muscle was found to be inappropriately active in 80% and the infraspinatus hypoactive in 25% of the shoulders with posterior subluxations or dislocations. Furthermore, they discovered that resisted external rotation and abduction during elevation of the arm prevents posterior subluxation of the arm [8]. This observation also explains why muscle stimulation of the infraspinatus muscle, as performed during our trial, can re-establish stability in patients with functional posterior instability. Activation of the external rotators leads to an anterior translation of the humeral head and prevents posterior subluxation or dislocation. Scapular dyskinesis, which is often present in patients with recurrent shoulder instability, leads to protraction of the scapula and a downward rotated position of the glenoid with decreased humeral head compression and stability [6, 10]. Therefore, additional stimulation of the periscapular muscles was applied aiming to reduce protraction and to stabilize the scapula to the thorax.

In a previous study, Jaggi et al. presented a comprehensive rehabilitation program for shoulder instability, which consists of an early, protective phase, followed by an intermediate phase and finally functional rehabilitation exercises [7]. For functional instability patients, the authors recommend exercises to increase the tone in postural muscles (core stabilization), which respectively reduces activity of the latissimus dorsi muscle and therefore the instability associated with posterior translation of the humeral head. Core stabilization exercises may then be followed by selective rotator cuff strengthening exercises with emphasis on the external rotators.

Takwale et al. established a treatment plan for patients with functional instability of the shoulder [23]. Their program aimed to retrain muscle patterns. At first, careful examination of the pathology was conducted, then the abnormal pattern of movement and muscle groups involved were analyzed. Finally, the patients were taught to recognize the inappropriate patterns and re-educated by applying tactile biofeedback techniques [23]. After a mean follow-up of 2 years, 52 out of 58 cases were graded as good to excellent. In six cases, poor results were observed and nine patients (12 shoulders) showed recurrent instability and required further episodes of retraining. However, in our experience an in-patient program with three to four sessions of treatment per day supervised by highly specialized physiotherapists is often not available to the general public, and improvements after standard physiotherapy in patients with functional shoulder instability are often small to nonexistent [23].

Therefore, we established this pilot project in order to evaluate how electric muscle stimulation in patients with functional shoulder instability can re-establish stability. The results show that electric stimulation of hypoactive external rotators and scapular retractors by means of the shoulder pacemaker seems to successfully re-establish stability in patients with functional shoulder instability during the time of application. Of course this pilot study lacks statistical power and gives no information on the short- or long-term effects of the shoulder pacemaker. However, it serves as valuable proof of concept for further investigations. In an upcoming prospective clinical trial, the shoulder pacemaker will be implemented in the regular physiotherapeutic treatment. The objective is to evaluate whether persistent shoulder stability can be achieved in patients with functional instability by physiotherapeutic treatment in combination with temporary application of electric muscle stimulation during training.

## Conclusion

Electrical stimulation of the rotator cuff and periscapular muscles seems to successfully confine functional shoulder instability with temporary but immediate and complete remission of clinical symptoms. Upcoming prospective clinical trials will show how an implementation of the shoulder pacemaker in a regular physiotherapeutic treatment program can lead to persistent shoulder stability and re-establish the muscular balance in affected patients.

## Caption Electronic Supplementary Material

Video showing the effect of the shoulder pacemaker when treating patients with functional shoulder instability.
